# Tools to identify linear combination of prognostic factors which maximizes area under receiver operator curve

**DOI:** 10.1186/2043-9113-4-10

**Published:** 2014-07-04

**Authors:** Nicolae Todor, Irina Todor, Gavril Săplăcan

**Affiliations:** 1 Oncology Institute "Prof.Dr. Ion Chiricuta", Biostatistics and Medical Informatics Department, Republicii 34-36, 400015 Cluj-Napoca, Romania; 2 Oncology Institute "Prof.Dr. Ion Chiricuta", Radiotherapy Department, Republicii 34-36, 400015 Cluj-Napoca, Romania; 3 Company for Applied Informatics, Republicii 101-102, 400015 Cluj-Napoca, Romania

**Keywords:** Area under curve, Linear combination, Receiver operator characteristics, Sensitivity, Specificity

## Abstract

**Background:**

The linear combination of variables is an attractive method in many medical analyses targeting a score to classify patients. In the case of ROC curves the most popular problem is to identify the linear combination which maximizes area under curve (AUC). This problem is complete closed when normality assumptions are met. With no assumption of normality search algorithm are avoided because it is accepted that we have to evaluate AUC n^d^ times where n is the number of distinct observation and d is the number of variables.

**Methods:**

For d = 2, using particularities of AUC formula, we described an algorithm which lowered the number of evaluations of AUC from n^2^ to n(n-1) + 1. For d > 2 our proposed solution is an approximate method by considering equidistant points on the unit sphere in R^d^ where we evaluate AUC.

**Results:**

The algorithms were applied to data from our lab to predict response of treatment by a set of molecular markers in cervical cancers patients. In order to evaluate the strength of our algorithms a simulation was added.

**Conclusions:**

In the case of no normality presented algorithms are feasible. For many variables computation time could be increased but acceptable.

## Background and previous results

In oncology one of the most used endpoint is treatment response. Let’s denote by *D* the associated variable. There are two possible values: *D* = 1 if the patient responds to treatment and *D* = 0 if the patient has no response.

Let’s suppose that there are two prognostic factors and let’s denote by *X*_1_ and *X*_2_ the random variable associated. *X*_1_ and *X*_2_ could be numeric or ordinal and the patient is getting better or worse as the value is smaller or bigger. For simplicity of the talk we suppose that both are numeric.

If *X* is one of *X*_1_ or *X*_2_ and *c* is a value from the range of *X* then the sensitivity (*Se*) or value "true positive" (TP) of variable *X* for *c* value is the probability that *X* > *c* for the patients which have a positive response to treatment:

$$Se_{X}\left(c\right)=TP_{X}\left(c\right)=P(X>c\left|D=1)\right)$$

The specificity (*Sp*) is the probability that *X* ≤ *c* for the patients which have no response "true negative" (TN):

$$Sp_{X}\left(c\right)=P(X\leq c\left|D=0)\right).$$

A special importance has "false postive" (*FP*) value defined by

$$FP_{X}\left(c\right)=1-Sp_{X}\left(c\right)=P(X>c\left|D=0)\right).$$

For a continuous variable *X*, "receiver operating characteristics" (ROC) curve [1] is the curve formed with the points

$$\left(1-SP_{X}\left(c\right),Se_{X}\left(c\right)\right)$$

that is

$$\left(FP_{X}\left(c\right),TP_{X}\left(c\right)\right)$$

for all possible values of *c*.

Area under curve (AUC) "measures" the potential influence of the random variable on treatment response. AUC values are between 0.5 and 1 and if they are in the proximity of 1 the variable is more important in the process of response prediction.

In the case of a discrete random variable with the numerical values *c*_1_ < *c*_2_ < … < *c*_*n*_, the ROC curve is formed by joining the points

$$\left\{\left(0,0\right),\left(\text{FP}\left(c_{1}\right),\text{TP}\left(c_{1}\right)\right),\left(\text{FP}\left(c_{2}\right),\text{TP}\left(c_{2}\right)\right),\ldots ,\left(\text{FP}\left(c_{n}\right),\text{TP}\left(c_{n}\right)\right),\left(1,1\right)\right\}$$

For continuous variables with unknown distributions the simplest way to evaluate AUC is to take a random sample and to build the polygonal line as for discrete variables.

The theory is similar if the signs > and < are changed each other in previous definitions. In practice it is chosen an increasing sequence *c*_1_ < *c*_2_ < … < *c*_*n*_or a decreasing sequence *c*_1_ > *c*_2_ > … > *c*_*n*_ so that AUC > 0.5.

Major interest is to test equality of AUC with 0.5.

If we have a unique random variable from all studied variables which has AUC > 0.5, at chosen significance level, than we can use this variable as prediction instrument.

If exists multiple variables with AUC > 0.5 emerges the problem of multivariate prediction counting on all variables.

Let’s suppose first that we have only two random variables. First natural variant is to choose a linear combination of the two variables as a global instrument of response prediction.

In formal terms the problem can be stated as an algorithm to find a pair of real numbers (*α*_1_, *α*_2_) so that global random variable

$$Z=\alpha_{1}X_{1}+\alpha_{2}X_{2}$$

induces a maximal AUC.

For a clear presentation let’s suppose that for the pair (*X*_1_, *X*_2_) there are *n* distinct observed values denoted by

(1) $$\left(x_{1i},x_{2i}\right),i=1,\ldots ,n$$

Also

(2) $$\left(n_{0i},n_{1i}\right),i=1,\ldots ,n$$

denote the number of patients that have no response, have response respectively for observation groups *i*;

(3) $$n_{0}=\sum_{i=1}^{n}n_{0i},n_{1}=\sum_{i=1}^{n}n_{1i}$$

denote the whole number of patients without response, with response respectively.

The ideea to solve frontal the problem without supplementary hyoptheses was generally rejected because at first sight the algorithms that evaluate AUC for all possible cases are complicated and this needs longer times to solve even for lower values of *n* and even with the help of computers.

Usually this problem is solved adding supplementary conditions or hypoteses to variables X_1_ and X_2_[2-8]. In [9,10] there are two comprehensive surveys. The problem is completly solved only when normality is supposed for variables X_1_ and X_2_. As sofware we have to mention SAS solution of [11] for normality case.

Present paper for a pair of variables (*X*_1_, *X*_2_) shows a reasonable algorithm which evaluates AUC for at most *n*(*n* - 1) + 1 times where *n* is the number of distinct values of the sample. For more than two variables it is proposed an algorithm which produces well aproximate solutions.

Firstly we prove some properties of linear combinations of two variables which are the basis of our algorithm. Next paragraph introduces an approximate solution for the case of two and extends the algorithm to more than two variables. An example occured in the cancer reaserch of our lab is presented subsequently. The example is solved with programs showed in Additional file 1. For each program short explanations or comments are inserted. The paper end with a summary of a simmulation on 20 studies with 200 observations each in order to evaluate the reliability of altgorithms.

## Results

### Properties of AUC evaluated for variables formed by linear combinations of two variables

The algorithm from next section is based on some elementary proprieties derived from the calculus formula of AUC.

Let’s suppose that there are two real values *α*_1_, *α*_2_ fixed and we try to evaluate AUC for the linear combination

*Z* = *α*_1_*X*_1_ + *α*_2_*X*_2_ with the observations shown at (1) and (2).

Let’s denote

(4) $$z_{i}=\alpha_{1}x_{1i}+\alpha_{2}x_{2i},i=1,\ldots ,n$$

the sample values of *Z* variable.

From [1,9,10,12] the formula to evaluate AUC for random variable *Z* is

(5) $$\text{AUC}=\frac{1}{n_{0}n_{1}}\sum_{i,j=1}^{n}n_{1i}n_{0j}\psi \left(z_{i},z_{j}\right)$$

where

(6) $$\psi \left(z_{i},z_{j}\right)=\left\{\begin{array}{l} 1\;\text{for}\;z_{i}>z_{j}\\ 0.5\;\text{for}\;z_{i}=z_{j}\\ 0\;\text{for}\;z_{i}<z_{j} \end{array}\right)$$

with *z*_1_, *z*_2_, …, *z*_*n*_ sorted ascending. In practice it is chosen ascending or descending order of *z*_1_, *z*_2_, …, *z*_*n*_ so that AUC ≥ 0.5 but the results are similar.

#### Property 1

For (*α*_1_, *α*_2_) fixed, ROC curve depends only by the order (increasing or decreasing) in which values *z*_1_, *z*_2_, …, *z*_*n*_ are.

**Proof** For fixed *α*_1_, *α*_2_ let’s denote *T*(*α*_1_, *α*_2_) = {*z*_*i*_ = *α*_1_*x*_1*i*_ + *α*_2_*x*_2*i*_|*i* = 1, …, *n*}

If *T*(*α*_1_, *α*_2_) has *m* distinct elements *t*_1_ < *t*_2_ < … < *t*_*m*_ and if *I*_*t*_ denotes the set of indexes so that the variable *Z* takes value *t*: *I*_*t*_ = {*i*|*z*_*i*_ = *t*} then from (5) and (6) ROC curve depends only by the set $M\left(\alpha_{1},\alpha_{2}\right)=\left\{I_{t_{1}},\ldots ,I_{t_{m}}\right\}$.

#### Property 2

Each point located on a line through origin determines same ROC curves.

**Proof** For a fixed pair*α*_1_, *α*_2_, {(*λα*_1_, *λα*_2_)|*λ* real, *λ* ≠ 0} is the line through origin. It produces same ROC curve due to fact that *M*(*λα*_1_, *λα*_2_) = *M*(*α*_1_, *α*_2_) for any *λ*.

#### Property 3

Each pair of values *i*_1_ ≠ *i*_2_ with $z_{i_{1}}=z_{i_{2}}$ determines a line trough origin and the points of this line generate same ROC curve.

**Proof** Let’s suppose that at least two values from the set *T*(*α*_1_, *α*_2_) are equal. Let’s denote *i*_1_, *i*_2_ two indexes with *i*_1_ ≠ *i*_2_ and $z_{i_{1}}=z_{i_{2}}$ that is

$$\alpha_{1}x_{1i_{1}}+\alpha_{2}x_{2i_{1}}=\alpha_{1}x_{1i_{2}}+\alpha_{2}x_{2i_{2}}$$

and further

(7) $$\alpha_{1}\left(x_{1i_{1}}-x_{1i_{2}}\right)+\alpha_{2}\left(x_{2i_{1}}-x_{2i_{2}}\right)=0$$

In the plane *α*_1_0*α*_2_, (7) is the equation of a line that passes through origin.

#### Property 4

The set of points (*α*_1_, *α*_2_) where ROC curve has same value is convex.

**Proof** The forth property shows that if $M\left(\alpha_{1},\alpha_{2}\right)=M\left(\alpha_{{}_{1}}^{'},\alpha_{{}_{2}}^{'}\right)$ for $\left(\alpha_{1},\alpha_{2}\right)\neq \left(\alpha_{{}_{1}}^{'},\alpha_{{}_{2}}^{'}\right)$ then $M\left(\alpha_{{}_{1}}^{"},\alpha_{{}_{2}}^{"}\right)=M\left(\alpha_{1},\alpha_{2}\right)=M\left(\alpha_{{}_{1}}^{'},\alpha_{{}_{2}}^{'}\right)$ for any point $\left(\alpha_{{}_{1}}^{"},\alpha_{{}_{2}}^{"}\right)$ located on the segment determined by (*α*_1_, *α*_2_) and $\left(\alpha_{{}_{1}}^{'},\alpha_{{}_{2}}^{'}\right)$. The proof comes from the observation that for any point $\left(\alpha_{{}_{1}}^{"},\alpha_{{}_{2}}^{"}\right)$ on the segment (*α*_1_, *α*_2_) and $\left(\alpha_{{}_{1}}^{'},\alpha_{{}_{2}}^{'}\right)$ there is a real number *λ* ∊ [0, 1] so that $\alpha_{{}_{1}}^{"}=\lambda \alpha_{1}+\left(1-\lambda \right)\alpha_{{}_{1}}^{'}$ and $\alpha_{{}_{2}}^{"}=\lambda \alpha_{2}+\left(1-\lambda \right)\alpha_{{}_{2}}^{'}$. We show that the order of values *z*_1_, *z*_2_, …, *z*_*n*_ remains unchanged also for $\left(\alpha_{{}_{1}}^{"},\alpha_{{}_{2}}^{"}\right)$.

Indeed, for two distinct indexes *i*, *j* with *z*_*i*_ < *z*_*j*_ for both (*α*_1_, *α*_2_) and $\left(\alpha_{{}_{1}}^{'},\alpha_{{}_{2}}^{'}\right)$ we compute the values of *z*_*i*_ and *z*_*j*_ for $\left(\alpha_{{}_{1}}^{"},\alpha_{{}_{2}}^{"}\right)$:

$$\begin{array}{l} z_{i}\left(\alpha_{{}_{1}}^{"},\alpha_{{}_{2}}^{"}\right)=\alpha_{{}_{1}}^{"}x_{1i}+\alpha_{{}_{2}}^{"}x_{2i}=\\ =\left(\lambda \alpha_{1}+\left(1-\lambda \right)\alpha_{{}_{1}}^{'}\right)x_{1i}+\left(\lambda \alpha_{2}+\left(1-\lambda \right)\alpha_{{}_{2}}^{'}\right)x_{2i}=\\ =\lambda \left(\alpha_{1}x_{1i}+\alpha_{2}x_{2i}\right)+\left(1-\lambda \right)\left(\alpha_{{}_{1}}^{'}x_{1i}+\alpha_{{}_{2}}^{'}x_{2i}\right)=\\ =\lambda z_{i}\left(\alpha_{1},\alpha_{2}\right)+\left(1-\lambda \right)z_{i}\left(\alpha_{{}_{1}}^{'},\alpha_{{}_{2}}^{'}\right)\\ \;<\lambda z_{j}\left(\alpha_{1},\alpha_{2}\right)+\left(1-\lambda \right)z_{j}\left(\alpha_{{}_{1}}^{'},\alpha_{{}_{2}}^{'}\right)=\\ =\lambda \left(\alpha_{1}x_{1j}+\alpha_{2}x_{2j}\right)+\left(1-\lambda \right)\left(\alpha_{{}_{1}}^{'}x_{1j}+\alpha_{{}_{2}}^{'}x_{2j}\right)=\\ =\left(\lambda \alpha_{1}+\left(1-\lambda \right)\alpha_{{}_{1}}^{'}\right)x_{1j}+\left(\lambda \alpha_{2}+\left(1-\lambda \right)\alpha_{{}_{2}}^{'}\right)x_{2j}=\\ =\alpha_{{}_{1}}^{"}x_{1j}+\alpha_{{}_{2}}^{"}x_{2j}=z_{j}\left(\alpha_{1}^{"},\alpha_{2}^{"}\right). \end{array}$$

Further if the order of *z*_1_, *z*_2_, …, *z*_*n*_ is unchanged for $\left(\alpha_{{}_{1}}^{"},\alpha_{{}_{2}}^{"}\right)$, the ROC curves are identical.

### Algorithm to identify the linear combination of two variables which maximizes AUC

We have to identify in plane (*α*_1_, *α*_2_) the regions where AUCs are constants. From previous section we know that these regions are infinite triangles with the peak in origin. These triangles can be defined by the lines coming from (7). The whole number of them is $C_{n}^{2}=\frac{n\left(n-1\right)}{2}$ and they divide the plane in maximum *C*_*n*_^2^ + 1 distinct regions. From the last property *M*(*α*_1_, *α*_2_) is constant if (*α*_1_, *α*_2_) are in the same region. Now we have to compute AUC for a point from each region and for a point from each line through origin that split two regions. The maximum number of AUC evaluations are *n*(*n* - 1) + 1.

To finish we need a strategy to chose the points where AUC will be evaluated. Our proposition consists of building up an auxiliary line that intersects all lines (7). The intersections with lines (7) generates maximum *C*_*n*_^2^ - 1 finite segments and two infinite segments. For the finite segments we have chosen the margins and the middles as points to evaluate AUC. For the infinite segments we have chosen points located at distance of one unit from the fixed margin.

The authors have a program in Additional file 1 by which they solved the problem from above. In this program they have chose for auxiliary line the slope equal with

(8) $$\underset{i,j=1,\ldots ,n}{\text{min}}\left\{\frac{x_{1j}-x_{1i}}{x_{2j}-x_{2i}}\left|i\neq j,x_{2j}\neq x_{2i}\right)\right\}-1$$

and the line passes through the point (0, 1). This slope is lower than all slopes derived from equations (7) so that the intersection points are certain.

Supplementary the points where we evaluate AUC can be chosen normalized conform to second property on the unity circle so that *α*_1_^2^ + *α*_2_^2^ = 1.

### Approximate methods to identify the linear combination with maximal AUC

In [5] the evaluation of (*α*_1_, *α*_2_) with *α*_2_ ≠ 0 in the expression *α*_1_*X*_1_ + *α*_2_*X*_2_ is reduced at the identification of *α* ∊ [ - 1, 1] in *X*_1_ + *αX*_2_ and then the interval [ - 1, 1] is divided in 201 equal segments. The maximal value is from the set of AUC on each segment extremity. Our proposition is to consider on unity circle all the points where AUC is evaluated. Supplementary from symmetry we need to evaluate AUC only in quadrant I and IV. More exactly we evaluate AUC for (*α*_1_, *α*_2_) with

$$\begin{array}{ll} \;\alpha_{1} & =\sin\theta ,\alpha_{2}=\cos\theta \;\mathrm{for}\;\theta \\ & =-\frac{\pi }{2},-\frac{\pi }{2}+\frac{\pi }{200},-\frac{\pi }{2}+2\frac{\pi }{200},\ldots ,-\frac{\pi }{2}+200\frac{\pi }{200}. \end{array}$$

The precision can be improved by dividing quadrant I and IV in more and more regions subsequently. Practically we divide the quadrant I and IV till the divisions are smaller than an apriori limit.

This view permits easy extension when we have more than two prognostic factors.

For *X*_1_, *X*_2_, …, *X*_*f*_ prognostic factors, with *f* > 2, extension consists in a method to highlight or to move on the unit sphere in space with *f* dimensions. Our proposal is to consider for *α*_1_, *α*_2_, …, *α*_*f*_ the following values:

(9) $$\left\{\begin{array}{l} \alpha_{1}=\cos\theta_{1}\\ \alpha_{2}=\sin\theta_{1}\cos\theta_{2}\\ \alpha_{3}=\sin\theta_{1}\sin\theta_{2}\cos\theta_{3}\\ \ldots \\ \alpha_{f-1}=\sin\theta_{1}\sin\theta_{2}\ldots \cos\theta_{f-1}\\ \alpha_{f}=\sin\theta_{1}\sin\theta_{2}\ldots \sin\theta_{f-1} \end{array}\right)$$

with

(10) $$\theta_{1},\ldots ,\theta_{f-1}\in \left\{-\frac{\pi }{2},-\frac{\pi }{2}+\frac{\pi }{200},-\frac{\pi }{2}+2\frac{\pi }{200},\ldots ,-\frac{\pi }{2}+200\frac{\pi }{200}\right\}.$$

Of course if we want to increase the precision we can increase the number points inside the interval $\left[-\frac{\pi }{2},+\frac{\pi }{2}\right]$.

The authors have a program in Additional file 1 which was used to solve the example from next section.

### Example

In [13] there is an interim result of a study for several molecular markers in relation to response to treatment for cervix cancers. Endpoint was considered the patient status found at 30 days after the end of treatment. We have *D* = 1 or *D* = 0 as the patient presented complete remission or residual tumor at 30 days. It were 14 patients with *D* = 1 and 12 patients with *D* = 0.

From univariate analysis were retained: Vascular Endothelial Growth Factor Receptor (VEGFR) (AUC = 0.74, p = 0.02), dimesion of tumor (AUC = 0.73, p = 0.001) and age (AUC = 0.67, p = 0.06). Logistic model for multivariate analysis [14] did not validate any linear combination of these factors.

Due to this failure we built a program associated to the method described in paragraph 3 (see Additional file 1).

We started by dividing quadrant I and IV in 50 parts. Linear combination that maximizes the AUC for this division has solution:

{0.998027, -0.0608178, 0.0156154}

and AUC = 0.815476.

Dividing the I-st and IV-th quadrant in 100 parts yields the following solution

{0.998027, -0.0602973, 0.017518},

{0.998027, -0.0608178, 0.0156154},

{0.995562, -0.0939226, 0.00590911}

and AUC = 0.815476.

For 150 parts the solution is

{0.998027, -0.0604775, 0.0168856},

{0.998027, -0.0608178, 0.0156154},

{0.996493, -0.0835127, 0.00525418}

and AUC = 0.815476

For 200 parts the solution is

{0.996917, -0.0753438, 0.0218894},

{0.996917, -0.0756783, 0.0207032}

and AUC = 0.821429.

For 300 parts the solution is

{0.997314, -0.0694128, 0.02336},

{0.997314, -0.0696537, 0.0226318},

{0.997314, -0.0698868, 0.0219012},

{0.997314, -0.0701124, 0.0211682},

{0.997314, -0.0714744, 0.0159764},

{0.997314, -0.0716378, 0.0152271}

and AUC = 0.821429.

As can be seen increased number of divisions for 50, 100 and 150 does not change the maximum of AUC but increases the number of points where maximum AUC value is reached.

**Figure 1:**
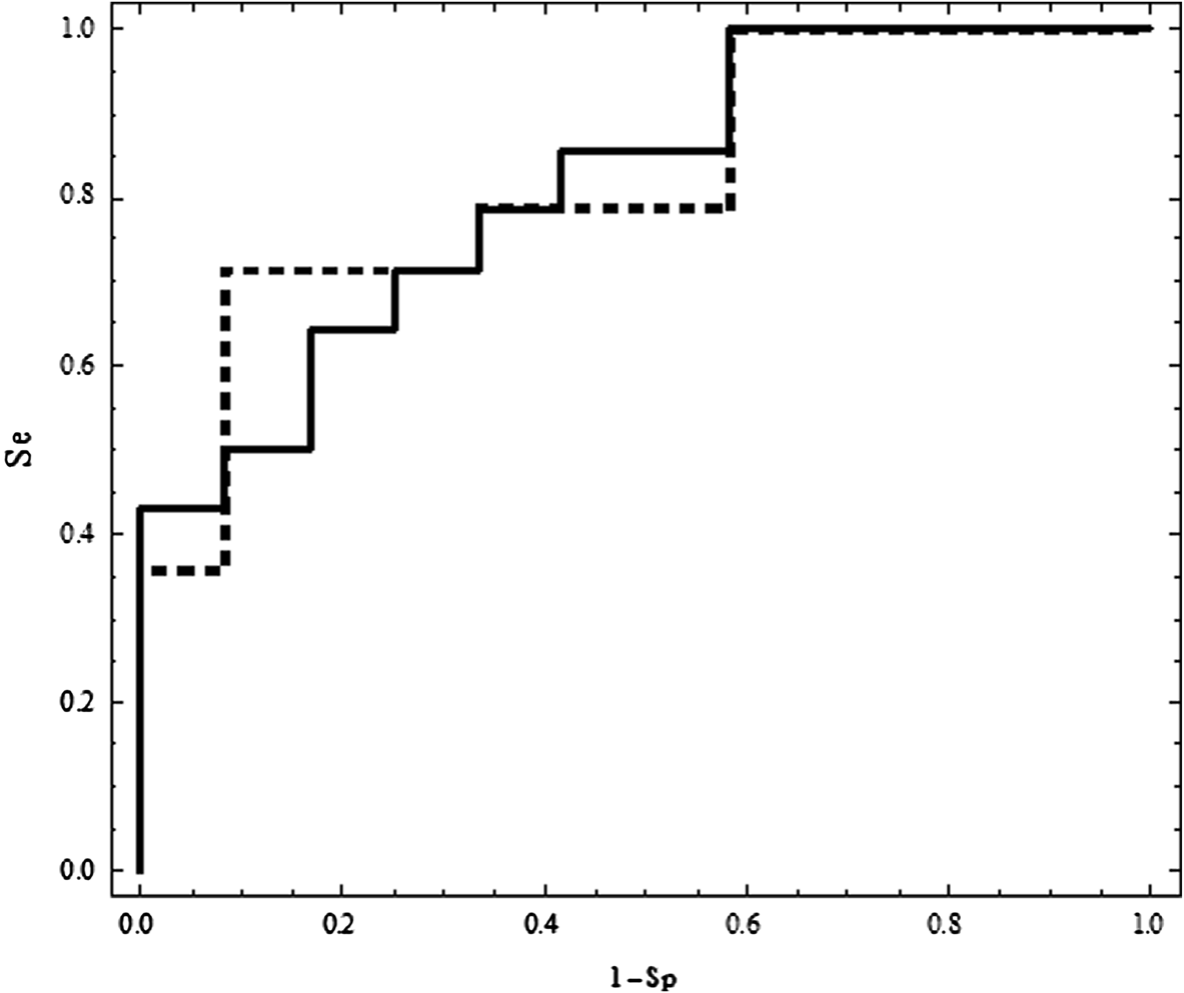
**ROC curves for score *0.998027*** **×** ***VEGFR*** **-** ***0.0608178*** **×** ***dimension of tumor*** **+** ***0.0156154*** **×** ***age *with AUC = 0.815476 and p = 0.000093 for 150 divisions (continuos line) and for score *0.996917*** **×** ***VEGFR*** **-** ***0.0753438*** **×** ***dimension of tumor*** **+** ***0.0218894*** **×** ***age *with AUC = 0.821429 and p = 0.000056 for 300 divisions (dashed line).**

For 200 and 300 divisions the same area under the curve with very small increase for AUC of 0.00595238 makes us believe that we are close to global solution.Figure 1 shows the ROC curves for the two linear combinations that give the two AUC values outlined above. We used firstly the score:

$$\begin{array}{ll} \;0.998027 & \times \mathrm{VEGFR}-0.0608178\times \mathrm{dimension}\;\mathrm{of}\;\mathrm{tumor}\\ & +0.0156154\times \mathrm{age} \end{array}$$

resulting from algorithm with 150 divisions then the score

$$\begin{array}{ll} \;0.996917 & \times \mathrm{VEGFR}-0.0753438\times \mathrm{dimension}\;\mathrm{of}\;\mathrm{tumor}\\ & +0.0218894\times \mathrm{age} \end{array}$$

resulting from algorithm with 300 divisions.

Note that both scores have the values of p highly significant, and we propose the solution that has higher AUC.

Although computer times were acceptable (between 37 seconds to 50 divisions and 1 hour and 13 minutes to 300 divisions) we do not believe that would be necessary to go further with the number of divisions and we believe that a good solution could be

$$\begin{array}{ll} \;0.996917 & \times \mathrm{VEGFR}-0.0753438\times \mathrm{dimension}\;\mathrm{of}\;\mathrm{tumor}\\ & +0.0218894\times \mathrm{age}. \end{array}$$

Furthermore criteria of classification from ROC curve analysis [9] tells us for this choice that patients with score higher than 1.425782 are patients from whom we expect a better result (Se = 0.71, Sp = 0.92).

### Simulation

As previous example has a small number of observations we have made a simulation for 20 studies with 200 observations each with three prognostic factors. For the first factor, cases were selected from a pseudonormal variable with mean 1 and standard deviation of 3 and controls from a pseudonormal variable with mean 3 and standard deviation 3.5. The second and third prognostic factor, also come from a pseudonormal variable with standard deviation of 3 and 3.5 respectively for cases and controls and with averages of 4 and 6 for controls respectively 6 and 6.5 for cases.

The simulation was made on a Lenovo computer with operating system Windows 7 Ultimate on 64-bit with an i7 processor at 1.37 Gz in parallel with the current work of the author i.e. text editing, Internet browsing and reading emails. The result of the simulation for the algorithm presented before for 50, 100 and 200 segments are shown in Table 1. It is noted that the jump from 50 to 100 segments produces a change in AUC only to the third decimal place (the maximum value of 0.0012 to simulation 15). Jump from 100 segments to 200 segments changes AUC only at the fourth decimal place (the maximum value of 0.0008 to simulation 14). We believe that in practice there is no need to move beyond 200 divisions only for outstanding situations.

**Table 1:** Results of 20 simulations with 200 observations

**Crt.Nb.**	**Time**	**AUC50**	**Time**	**AUC100**	**Time**	**AUC200**	**AUC100 - AUC50**	**AUC200 - AUC100**
1	1314s (0H 21M 54 s)	0.7091	5033 s (1H 23M 53 s)	0.7098	20354 s (5H 39M 14 s)	0.7098	0.0007	0.0000
2	1283s (0H 21M 23 s)	0.6589	5154 s (1H 25M 54 s)	0.6589	31636 s (8H 47M 16 s)	0.6589	0.0000	0.0000
3	1501s (0H 25M 1 s)	0.6406	5842 s (1H 37M 22 s)	0.6412	23352 s (6H 29M 12 s)	0.6412	0.0006	0.0000
4	1173s (0H 19M 33 s)	0.6862	4681 s (1H 18M 1 s)	0.6862	25012 s (6H 56M 52 s)	0.6867	0.0000	0.0005
5	1277s (0H 21M 17 s)	0.6629	10790 s (2H 59M 50s)	0.6633	12321 s (3H 25M 21 s)	0.6638	0.0004	0.0005
6	1353s (0H 22M 33 s)	0.6715	4574 s (1H 16M 14 s)	0.6717	15292 s (4H 14M 52 s)	0.6726	0.0002	0.0009
7	1342s (0H 22M 22 s)	0.6761	5132 s (1H 25M 32 s)	0.6772	18625 s (5H 10M 25 s)	0.6773	0.0011	0.0001
8	1297s (0H 21M 37 s)	0.6944	6988 s (1H 56M 28 s)	0.6953	18813 s (5H 13M 33 s)	0.6954	0.0009	0.0001
9	1070s (0H 17M 50s)	0.6988	5399 s (1H 29M 59 s)	0.6990	19498 s (5H 24M 58 s)	0.6994	0.0002	0.0004
10	536 s (0H 8M 56 s)	0.6638	3022 s (0H 50M 22 s)	0.6640	18556 s (5H 9M 16 s)	0.6646	0.0002	0.0006
11	1329s (0H 22M 9 s)	0.6900	4766 s (1H 19M 26 s)	0.6902	20419 s (5H 40M 19 s)	0.6906	0.0002	0.0004
12	1288s (0H 21M 28 s)	0.6946	5086 s (1H 24M 46 s)	0.6948	20573 s (5H 42M 53 s)	0.6948	0.0002	0.0000
13	637 s (0H 10M 37 s)	0.6873	2454 s (0H 40M 54 s)	0.6875	21271 s (5H 54M 31 s)	0.6875	0.0002	0.0000
14	513 s (0H 8M 33 s)	0.7031	2025s (0H 33M 45 s)	0.7032	20139 s (5H 35M 39 s)	0.7040	0.0001	0.0008
15	952 s (0H 15M 52 s)	0.7200	2082s (0H 34M 42 s)	0.7202	21224 s (5H 53M 44 s)	0.7204	0.0002	0.0002
16	1176s (0H 19M 36 s)	0.7401	4923 s (1H 22M 3 s)	0.7413	27836 s (7H 43M 56 s)	0.7413	0.0012	0.0000
17	796 s (0H 13M 16 s)	0.7398	4332 s (1H 12M 12 s)	0.7399	18213 s (5H 3M 33 s)	0.7405	0.0001	0.0006
18	1296s (0H 21M 36 s)	0.6635	2534 s (0H 42M 14 s)	0.6638	20165 s (5H 36M 5 s)	0.6644	0.0003	0.0006
19	797 s (0H 13M 17 s)	0.7041	3407 s (0H 56M 47 s)	0.7045	20313 s (5H 38M 33 s)	0.7045	0.0004	0.0000
20	1420s (0H 23M 40s)	0.6825	5532 s (1H 32M 12 s)	0.6826	15051 s (4H 10M 51 s)	0.6826	0.0001	0.0000
Average	1117s (0H 18M 37 s)		4687 s (1H 18M 7 s)		20433 s (5H 40M 33 s)			

AUC50, AU C100, AUC200 denotes the approximation of AUC by dividing interval $\left[-\frac{\pi }{2},+\frac{\pi }{2}\right]$ in 50, 100, 200 equal parts.

Average execution time for 50, 100 and 200 segments was 18 minutes, 1 hour and 18 minutes, 5 hours and 40 minutes which is an acceptable time for a practical problem.

## Discussions and conclusions

Multivariate analysis is used largely in any medical paper. However testing the hypotheses in modeling is not a very simple task and this is the reason for trying a lot of potential models and choose the model best suited to observations. The papers of [4,6,15-19] prove that there is a large basis to use linear combinations of variables in ROC analysis. If we do not have solid condition to apply for example one of the cited models, the method from our paper produces always a score for which we have maximal AUC or an approximate.

On the other hand in a classical model of regression it is known that the numerical methods used to identify the model parameters not always provide a global maximum and depends heavily on the initial values of the algorithm. The solution presented we believe could be used there as a baseline for these algorithms.

The main advantage of presented algorithms is that it always provides a solution. However for many prognostic factors and observations, time of the calculation could be a problem.

Certainly, approximate method is more appropriate in this last case despite the fact that it does not guarantee a global solution. However it is guaranteed to yield a solution with AUC higher than each variable taken separately.

Our algorithm can be used in any medical paper as an alternate method for multivariate analysis.

The presented algorithms have major advantage to provide always a solution with no supplementary constraints.

For many variables computation time is high but not high enough as not to accept this cost.

## Competing interests

The authors declare that they have no competing interests.

## Authors’ contributions

Authors have equal contributions but main responsibilities were as follows. NT has the idea of the study, and participated in its design and coordination. IT carried out the medical data, performed the statistical analysis and drafted the manuscript. SG carried out programming and design of the algotithms. All authors read and approved the final manuscript.

## Supplementary Material

Additional file 1 AUC evaluation, Maximum AUC evaluation for a pair of variables, Maximum AUC evaluation for more than two variables. Click here for file
