# Supplementary material for: Tools to identify linear combination of prognostic factors which maximizes area under receiver operator curve
Source: J Clin Bioinforma. 2014 Jul 4;4:10. doi: 10.1186/2043-9113-4-10 (PMC4099021; doi:10.1186/2043-9113-4-10)
Supplement: Additional file 1 — AUC evaluation, Maximum AUC evaluation for a pair of variables, Maximum AUC evaluation for more than two variables. [file 2043-9113-4-10-S1.doc]

Additional file 1

**A.1 AUC evaluation**

The *Mathematica* program form below evaluates AUC for observations presented in the sequences var and status. The output is a pair of values containing AUC and symbol < or > as the sequence of value from var is sorted ascending or descending in order to be sure that AUC is greater than 0.5.

_______________________________________________________________________

AUC2[var_, status_] :=

Module[{dir = ">", dt = {}, i, nr0 = Count[status, 0], nr1 = Count[status, 1],

rez = 0., nobs = Length[status]},

(*var is the sequence of observed values that contains numerical data*)

(*status is the sequence of observed status values each value is 0 *)

(*or 1 as the patient is alive or dead*)

(*the output is the pair of values {AUC,dir} where:*)

(* the values from var are sorted ascending or descending so that AUC *)

(* is superior of 0.5 *)

(* dir is a string that contains symbols < or > *)

(* as the values of var were sorted ascending or descending *)

For[i = 1, i <= Length[var], i++,

dt = Append[dt, {var[[i]], status[[i]]}];

];

(* we start with dir='>' *)

For[i = 1, i <= nobs, i++,

For[j = 1, j <= nobs, j++,

If[dt[[i, 2]] == 0 && dt[[j, 2]] == 1,

rez = rez + If[dt[[i, 1]] > dt[[j, 1]], 1, If[dt[[i, 1]] == dt[[j, 1]], 0.5, 0.]];

(* rez=rez+{1 or 0 or 0.5} *)

];

];

];

rez = rez/(nr0 nr1);

If[rez < 0.5,

(* dir='<' if AUC<0.5 *)

rez = 0; dir = "<";

For[i = 1, i <= nobs, i++,

For[j = 1, j <= nobs, j++,

If[dt[[i, 2]] == 0 && dt[[j, 2]] == 1,

rez = rez + If[dt[[j, 1]] > dt[[i, 1]], 1, If[dt[[j, 1]] == dt[[i, 1]], 0.5, 0.]];

(* rez=rez+{1 or 0 or 0.5} *)

];

];

];

rez = rez/(nr0 nr1);

];

{rez, dir}

]

_______________________________________________________________________

The following example evaluate AUC for variable VEGFR [12].

(* VEGFR *)

VEGFR = {0.0780206593063508, 0.146604368653985, 0.12413656192963, 0.19888412093873, 0.238159499510984, 0.343885454534936, 0.30145195692269, 0.264254510140345, 0.426317445883979, 0.366021423986407, 0.403320879611064, 0.417543959714185, 0.3789291416276, 0.539614118252215, 0.524858341811534, 0.521232880420562, 0.611320138846035, 0.726986258660155, 0.78458409789675, 0.7791645796605, 0.829319545814442, 0.913831450229403, 0.858565436437754, 0.965936328924848, 1.60213975517925, 1.81503831063432};

status = {0, 0, 0, 1, 1, 1, 0, 0, 1, 0, 1, 0, 0, 0, 0, 1, 1, 1, 1, 0, 1, 1, 0, 1, 1, 1};

Print["AUC[VEGFR]=", AUC2[VEGFR, status], "; AUC2[-VEGFR]=", AUC2[-VEGFR, status]]

with the output

AUC[VEGFR]={0.738095,<}; AUC2[-VEGFR]={0.738095,>}

As it can be seen AUC is equal for VEGFR and - VEGFR.

**A.2 Maximum AUC evaluation for a pair of variables**

The *Mathematica* program form below evaluates maximum AUC for a pair of variables as was shown an section 2. The variables are denoted by A and B and “status” has same definition as in appendix 1. The program is divided in segments (cells in Mathematica) in order to understand better the flow of information and to monitor better. Each module is over commented inside the lines of the program and followed of the output of the execution for control. At run time it is recommended to execute each module separately. The numerical examples of variables are from [12].

_______________________________________________________________________

(* here we have to place the code of routine AUC2 from appendix 1 *)

(* first variable, variable A in the example from below represent the dimension of tumor at start of treatment *)

A = {2.5, 2, 6, 4.5, 5, 4, 5, 7, 2, 4.5, 4.5, 5.5, 6, 3, 4, 6.5, 2, 4, 2.5, 5, 5, 4, 7, 2, 5, 5};

(* second variable age at the start of treatment *)

B = {51, 56, 28, 53, 73, 59, 56, 44, 55, 62, 68, 36, 41, 59, 55, 45, 47, 54, 56, 35, 28, 40, 50, 53, 57, 58};

(* for each observation status is 0 or 1 as the patient is alive or dead *)

status = {0, 0, 0, 1, 1, 1, 0, 0, 1, 0, 1, 0, 0, 0, 0, 1, 1, 1, 1, 0, 1, 1, 0, 1, 1, 1};

(* for control the AUC for A and B is printed *)

Print["A=", AUC2[A, status], "; B=", AUC2[B, status]]

_______________________________________________________________________

{A= {0.6636904761904762, ">"}, "; B=", { 0.6279761904761905, "<"}]

_______________________________________________________________________

(* gr contains parametric representation of the lines (7) from §2 formed by all pairs of observations from variable A and B *)

(* mnm at the end represents the value from (8) *)

gr = {}; mnm = -1;

For[i = 1, i < Length[A], i++,

For[j = i + 1, j <= Length[A], j++,

If[B[[i]] == B[[j]],

{

gr = Append[gr, {0, t}]

}, {

gr = Append[gr, {t, (A[[j]] - A[[i]])/(B[[j]] - B[[i]]) t}];

If[mnm > (A[[j]] - A[[i]])/(B[[j]] - B[[i]]), mnm = (A[[j]] - A[[i]])/(B[[j]] - B[[i]])]

}

]

]

]

mnm = mnm - 1;

gr = Append[gr, {t, mnm t + 1}];

(* a plot of all lines are produced only for monitoring*)

(* the plot can be omitted in order to increase the speed *)

ParametricPlot[gr, {t, -1, 1}]

_______________________________________________________________________

The next module evaluate the x-coordinate of the crosses of lines (7) with the auxiliary line from the end of §2.

_______________________________________________________________________

(* margins of the regions are considered firstly *)

(* at the end xUri contains all x-coordinates where AUC will be evaluated *)

xUri = {};

For[i = 1, i < Length[A], i++,

For[j = i + 1, j <= Length[A], j++,

If[B[[i]] == B[[j]],

xUri = Append[xUri, 0],

xUri = Append[xUri, 1./((A[[j]] - A[[i]])/(B[[j]] - B[[i]]) - mnm)]

]

]

]

(* if you want to see the values of mnm and xUri you can omit ; at the end of the line *)

mnm;

xUri;

(* double points are omitted by this Union *)

xUri = Union[xUri];

xUri2 = {xUri[[1]] - 1};

(* middle of the regions are added as points to evaluate AUC *)

For[i = 1, i < Length[xUri], i++,

xUri2 = Append[xUri2, xUri[[i]]];

xUri2 = Append[xUri2, (xUri[[i]] + xUri[[i + 1]])/2.];

]

xUri2 = Append[xUri2, Last[xUri]];

xUri2 = Append[xUri2, Last[xUri] + 1];

xUri2;

_______________________________________________________________________

The next module compute AUC for the point computed in the previous section. A matrix with maximum values is printed.

_______________________________________________________________________

(* auc2 will contain all AUC values computed *)

auc2 = {};

vMax = 0;

For[i = 1, i <= Length[xUri2], i++,

v1 = xUri2[[i]];

v2 = mnm v1 + 1;

var = v1 A + v2 B;

vAUC = AUC2[var, status];

If[vAUC[[1]] > vMax, vMax = vAUC[[1]]];

auc2 = Append[auc2, {i, v1, v2, vAUC}];

];

(* if someone want to see all values ; should be put away at the end of next line *)

MatrixForm[auc2];

(* position and maximum values will be put in auc4 *)

vMax;

auc4 = {};

For[i = 1, i <= Length[auc2], i++,

If[vMax == auc2[[i, 4]][[1]], auc4 = Append[auc4, auc2[[i]]]]

]

MatrixForm[auc4]

_______________________________________________________________________

169 0.183729 -0.0105074 {0.684524,>}

170 0.183784 -0.0108108 {0.684524,>}

171 0.183804 -0.0109201 {0.684524,>}

172 0.183824 -0.0110294 {0.684524,>}

173 0.183866 -0.0112618 {0.684524,>}

A graphic is plotted in order to see all possible values as final result. It can be omitted.

**_______________________________________________________________________**

auc3 = {};

For[i = 1, i <= Length[auc2], i++,

auc3 = Append[auc3, {auc2[[i, 2]], auc2[[i, 4]][[1]]}];

]

ListPlot[auc3, PlotRange -> {0, 1}, Joined -> True]

auc3;

_______________________________________________________________________

**A.3 Maximum AUC evaluation for more than two variables**

The *Mathematica* program from below evaluates maximum AUC for more than two variables following the algorithm suggested in section 3 and section 4.2. The database is coming from [12].

_______________________________________________________________________

(* here we have to place the code of routine AUC2 from appendix 1 *)

(* pas represents the number of segments in which the quadrant I and IV is divided *)

pas = 51

(* represents how many dimensions are used in the algorithm. 3 means that there are 3 variables and we are interested to identify a linear combination with 3 variables so that AUC is maximal *)

n = 3

n2 = n – 1

(* status as previous *)

status = { 0, 0, 0, 1, 1, 0, 0, 1, 0, 0, 1, 0, 1, 1, 0, 0, 1, 1, 0, 1, 1, 0, 1, 1, 1, 1 };

(* dPrim represents the observations. Each element has n components representing the variables of interest *)

dPrim = { {0.0780206593063508, 2.5, 51}, {0.12413656192963, 6, 28}, {0.146604368653985, 2, 56}, {0.19888412093873, 4.5, 53}, {0.238159499510984, 5, 73}, {0.264254510140345, 7, 44}, {0.30145195692269, 5, 56}, {0.343885454534936, 4, 59}, {0.366021423986407, 4.5, 62}, {0.3789291416276, 6, 41}, {0.403320879611064, 4.5, 68}, {0.417543959714185, 5.5, 36}, {0.426317445883979, 2, 55}, {0.521232880420562, 6.5, 45}, {0.524858341811534, 4, 55}, {0.539614118252215, 3, 59}, {0.611320138846035, 2, 47}, {0.726986258660155, 4, 54}, {0.7791645796605, 5, 35}, {0.78458409789675, 2.5, 56}, {0.829319545814442, 5, 28}, {0.858565436437754, 7, 50}, {0.913831450229403, 4, 40}, {0.965936328924848, 2, 53}, {1.60213975517925, 5, 57}, {1.81503831063432, 5, 58} }

_______________________________________________________________________

The next module is the workhorse of the algorithm

_______________________________________________________________________

coef2 = {};

maxAUC = -1;

valAUC = {};

valAUC3 = {};

For[i = 0, i < pas^n2, i++,

teta = {};

kj = i;

For[j = 1, j <= n2, j++,

kj2 = Mod[kj, pas];

kj = (kj - kj2 )/ pas;

teta = Append[teta, kj2];//calculus of teta as in paragraph 4 and (10)

];

teta = -Pi/2 + 2 teta Pi/(2 (pas - 1));

coef = Table[1, {k, 1, n}];

For[j = 1, j <= n2, j++,

coef[[j]] = coef[[j]] Cos[N[teta[[j]]]];

];

For[j = 2, j <= n, j++,

For[k = j, k <= n, k++,

coef[[k]] = coef[[k] ] Sin[N[teta[[j - 1]]]];// coefficients as in (9)

];

];

score = {};

For[j = 1, j <= Length[dPrim], j++,

score2 = 0;

For[k = 1, k <= n, k++,

score2 = score2 + N[dPrim[[j, k]]*coef[[k]]];

];

score = Append[score, score2];

];

valAUC2 = AUC2[score, status];

valAUC3 = Append[valAUC3, valAUC2[[1]]];

valAUC = Append[valAUC, {valAUC2, coef}];

If[maxAUC < valAUC2[[1]], maxAUC = valAUC2[[1]]];

coef2 = Append[coef2, coef];

]

(* final results *)

maxAUC

(* matrix with results can be printed if you omit the ; at the end of next line. maybe the system will ask you to confirm the print of a large number of pages *)

MatrixForm[valAUC];

(* final solution is plotted to see where is the solution; it can be omitted to increase the speed *)

ListPlot[valAUC3]

(* sol contains the final solution. It is printed the AUC and the value associated of the variables*)

sol = {};

For[i = 1, i <= Length[valAUC], i++,

If[N[maxAUC] == N[valAUC[[i, 1, 1]]],

sol = Append[sol, valAUC[[i]]];];

];

MatrixForm[sol]

______________________________________________________________________

({0.815476,<} {0.998027,-0.0608178,0.0156154})
